# Gene-Trap Mutagenesis Identifies Mammalian Genes Contributing to Intoxication by *Clostridium perfringens* ε-Toxin

**DOI:** 10.1371/journal.pone.0017787

**Published:** 2011-03-11

**Authors:** Susan E. Ivie, Christine M. Fennessey, Jinsong Sheng, Donald H. Rubin, Mark S. McClain

**Affiliations:** 1 Division of Infectious Disease, Department of Medicine, Vanderbilt University School of Medicine, Nashville, Tennessee, United States of America; 2 Department of Microbiology and Immunology, Vanderbilt University School of Medicine, Nashville, Tennessee, United States of America; 3 Research Medicine, VA Tennessee Valley Healthcare System, Nashville, Tennessee, United States of America; Max Planck Institute for Infection Biology, Germany

## Abstract

The *Clostridium perfringens* ε-toxin is an extremely potent toxin associated with lethal toxemias in domesticated ruminants and may be toxic to humans. Intoxication results in fluid accumulation in various tissues, most notably in the brain and kidneys. Previous studies suggest that the toxin is a pore-forming toxin, leading to dysregulated ion homeostasis and ultimately cell death. However, mammalian host factors that likely contribute to ε-toxin-induced cytotoxicity are poorly understood. A library of insertional mutant Madin Darby canine kidney (MDCK) cells, which are highly susceptible to the lethal affects of ε-toxin, was used to select clones of cells resistant to ε-toxin-induced cytotoxicity. The genes mutated in 9 surviving resistant cell clones were identified. We focused additional experiments on one of the identified genes as a means of validating the experimental approach. Gene expression microarray analysis revealed that one of the identified genes, hepatitis A virus cellular receptor 1 (HAVCR1, KIM-1, TIM1), is more abundantly expressed in human kidney cell lines than it is expressed in human cells known to be resistant to ε-toxin. One human kidney cell line, ACHN, was found to be sensitive to the toxin and expresses a larger isoform of the HAVCR1 protein than the HAVCR1 protein expressed by other, toxin-resistant human kidney cell lines. RNA interference studies in MDCK and in ACHN cells confirmed that HAVCR1 contributes to ε-toxin-induced cytotoxicity. Additionally, ε-toxin was shown to bind to HAVCR1 *in vitro*. The results of this study indicate that *HAVCR1* and the other genes identified through the use of gene-trap mutagenesis and RNA interference strategies represent important targets for investigation of the process by which ε-toxin induces cell death and new targets for potential therapeutic intervention.

## Introduction

The *Clostridium perfringens* ε-toxin is responsible for a lethal enterotoxemia in livestock animals, and possibly in humans [1]. The U.S. Department of Health and Human Services has classified the ε-toxin as a select agent. Intoxication leads to increased permeability of the small intestine and ultimately causes widespread vascular permeability [2], [3], [4], [5], [6]. The toxin is believed to enter into the bloodstream and disseminate throughout the body where it accumulates primarily in the kidneys and brain of intoxicated animals [4], [7], [8]. Symptoms of ε-toxin intoxication typically indicate central nervous system involvement and may include incoordination, convulsions, or coma before death [9], [10], [11]. As is true of many select agents and toxins, human exposure to ε-toxin appears to be rare. In contrast to sheep and other livestock, humans are infrequently infected by *C. perfringens* strains capable of expressing ε-toxin [12], [13]. Studies do suggest, however, that ε-toxin may contribute to adverse health effects in humans. At least two case studies provide evidence of ε-toxin production in humans [14], [15], and additional case studies with diverse clinical outcomes have reported human infection by ε-toxin-producing strains of *C. perfringens* (e.g., [16], [17]). Many case studies of *C. perfringens* infection do not provide information concerning the toxins produced by the isolated strains. Although natural infection of humans by ε-toxin-producing *C. perfringens* is rare, weaponization of the purified ε-toxin could present the toxin at either higher doses and via routes of exposure not normally encountered and thus could present unique challenges to humans exposed to the toxin. No therapy to counteract ε-toxin is approved for use in humans.

Though detailed binding studies have not been reported, evidence from numerous studies suggests that ε-toxin binds to a specific receptor. The toxin is secreted from *C. perfringens* as a relatively inactive precursor or prototoxin. In mice, toxin binding to the brain is inhibited by prior administration of the inactive prototoxin [7], [18]. Similarly, binding of the toxin to isolated membranes is saturable and is inhibited by inactive ε-prototoxin [19]. Treatment of membrane fractions with pronase or neuraminidase decreases toxin binding, suggesting that a sialoglycoprotein is the cell-surface receptor [19]. However, the identity of the receptor remains to be determined.

The events leading to cell death in response to ε-toxin are not thoroughly understood, and multiple pathways of cell death may be involved. Addition of ε-toxin to MDCK cells leads to the formation of detergent-resistant toxin oligomers [20], [21], [22]. Formation of the oligomeric complexes is detectable as early as 15 minutes after toxin addition to MDCK cells, at which time 10 to 20% of the monolayer has been killed [22]. Formation of these oligomeric complexes is observed when ε-toxin is added to sensitive, but not resistant, cell lines [21]. In addition, the active form of ε-toxin, but not the inactive prototoxin, is able to form the detergent-resistant complexes [21]. Specifically, removal of a carboxy-terminal peptide from the ε-prototoxin upon activation is required for both the increased cytotoxicity and the ability to form oligomeric complexes [20]. Treating MDCK cells with ε-toxin is rapidly followed by efflux of intracellular K^+^ and increases in intracellular Cl^−^ and Na^+^ [22], [23]. There is no evidence that the ε-toxin enters cells [21], [22], [24]. Thus, in one pathway, the lethal activity of the toxin may be a direct effect of the toxin forming oligomeric pores in the plasma membrane of target cells, leading to depolarization of the cell's electrochemical gradient, disruption of ion homeostasis, and cell death. However, an alternate pathway leading to cell death also may be involved. Addition of ε-toxin to a murine renal cortical collecting duct cell line leads to a rapid depletion of cellular ATP levels, stimulates AMP-activated protein kinase, and induces nuclear translocation of apoptosis-inducing factor, a potent caspase-independent cell death effector [25]. In this latter study, the ATP-depletion and cell death appeared to be independent of toxin oligomerization and the formation of pores [25]. Thus host factors, in addition to the cell-surface receptor, may contribute to ε-toxin-mediated cytotoxicity.

A variety of studies exploring the cytotoxic activities of other pore-forming toxins suggest that host factors (beyond cell-surface receptors) also contribute to toxin-induced cytotoxicity [26], [27], [28], [29], [30], [31], [32], [33]. For example, the mammalian protein kinase A pathway has been shown to be required for Cry1Ab-induced cell death [31]. Additionally, *E. coli* α-hemolysin has been shown to lead to leakage of ATP from cells; the extracellular ATP then activates P2X pores that potentiate cell lysis [32]. Finally, pre-treatment of glioma cells with inhibitors of mitogen-activated/extracellular regulated kinase 1, protein kinase C, or Ca^2+^/calmodulin-dependent kinase II protects cells from Bc2 and equinatoxin-II [33]. These previous studies indicate that the mechanism by which pore-forming toxins mediate cell death is more complicated than previously thought and that inhibition of cellular responses can protect from cell death induced by pore-forming toxins. Thus, identifying host factors contributing to the activity of pore-forming toxins is expected to identify candidates for new therapeutic approaches. Previous studies have used forward genetic screens in hypodiploid cell lines to identify host factors involved in the activity of bacterial protein toxins [34], [35]. Although successful, the use of hypodiploid cell lines limits the utility of these approaches.

In the present study, we use gene trap selection in a diploid cell line to identify mammalian factors contributing to ε-toxin-induced cytotoxicity. Analysis of gene expression microarray data in human cell lines led us to focus on one of the identified genes, *HAVCR1*, and to the identification of a human cell line that is sensitive to ε-toxin. We confirmed a role for HAVCR1 in ε-toxin-induced cytotoxicity using shRNA in both canine and human cell lines and demonstrated that ε-toxin binds to HAVCR1 *in vitro*.

## Results

### Identification of mammalian genes by gene-trap mutagenesis and selection of ε-toxin-resistant MDCK cells

To identify mammalian host factors contributing to ε-toxin-induced cytotoxicity, we used a genetic selection to isolate toxin-resistant MDCK cells. Gene trap insertional mutagenesis involves infection of a mammalian cell with a replication-deficient retroviral vector that integrates its genetic material into the host cell chromosome with no known site specificity, carrying with it a promoterless neomycin resistance gene (neo^r^) [36]. Insertion of the gene trap vector into an actively transcribed region of the chromosome is expected to confer resistance to neomycin (G418) and to disrupt expression of the gene into which the vector has inserted. We have applied this approach to identify mammalian genes whose disruption confers increased resistance to challenge with purified ε-toxin.

**Figure 1:**
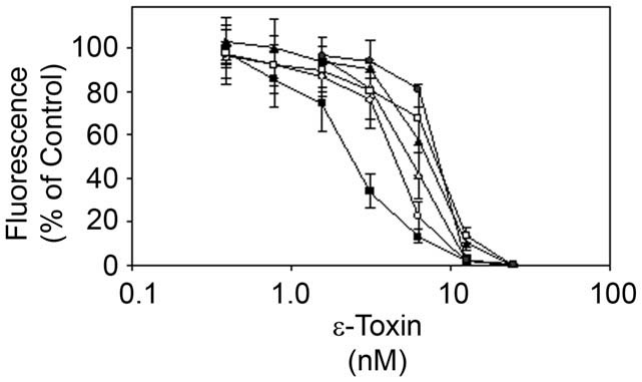
MDCK insertional mutants with increased resistance to ε-toxin. An MDCK cell gene trap library was treated with purified ε-toxin as described in Materials and Methods. Clonal populations of surviving cells were isolated, and treated with serial dilutions of purified ε-toxin. Cell viability was assessed as described in Materials and Methods. Results represent the mean and standard deviation of quadruplicate samples. To simplify the graphical display, results from wild-type MDCK (▪) and 5 representative mutant clones are displayed (□,CCDC134; •, HAVCR1; ○, SGMS2; ▴, XPO1; and ▵, ZBTB20); similar results were observed for the remaining clones. The toxin dose needed to kill 50% of each cell line was calculated by non-linear regression analysis, and values were compared by ANOVA followed by Dunnett's *post hoc* test. Each of the selected mutant cell lines required a greater amount of toxin to kill 50% of the cells than was required to kill 50% of the parental MDCK cells (P<0.05).

A gene trap library was generated in MDCK cells, a cell line widely used in studies of ε-toxin activity *in vitro* [37]. Following infection with the gene trap vector and selection of G418-resistant cells, the cells were incubated in medium containing ε-toxin as described in Materials and Methods. Cells that survived the toxin challenge were cloned, and the sensitivity of the cloned cell lines to ε-toxin was assessed by incubating the cells with serial dilutions of purified toxin (Figure 1). Though each of the selected mutant MDCK cells displayed increased resistance to epsilon toxin, none were as resistant as naturally-occurring resistant cell lines (data not shown). This may be due, at least in part, to expression from the non-mutated allele following gene-trap insertional mutagenesis (if the second allele is transcribed, then a reduction in expression, rather than full knockdown is anticipated, with a resultant diminution of toxin activity rather than full resistance to intoxication). It also is possible that the naturally-resistant cell lines lack multiple factors required for ε-toxin-induced cytotoxicity. The disrupted genes (identified as described in Materials and Methods) are listed in Table 1 and include the gene encoding sphingomyelin synthase 2, responsible for the last step in the synthesis of sphingomyelin. Previous studies have demonstrated that inhibition of sphingomyelin synthesis leads to increased resistance to ε-toxin [38].

**Table 1:** Genes identified in ε-toxin-resistant MDCK cells.

Gene Symbol^a^	Gene Name	Aliases
BCL3	B-cell CLL/lymphoma 3	D19S37, BCL4
CAV2	Caveolin 2	
CCDC134	Coiled-coil domain containing 134	FLJ22349
SMN^b^orDUSP5^b^	Survival of motor neuronDual specificity phosphatase 5	HVH3
HAVCR1	Hepatitis A virus cellular receptor 1	HAVCR-1, TIM-1, TIM1, HAVCR, TIMD1, KIM1
ZMYND8	Zinc finger, MYND-type containing 8	RACK7
SGMS2	Sphingomyelin synthase 2	MGC26963, SMS2
XPO1	Exportin 1	CRM1
ZBTB20	Zinc finger and BTB domain containing 20	ODA-8S, DKFZp566F123, DPZF

a Gene nomenclature is based on recommendations of the HUGO Gene Nomenclature Committee at the European Bioinformatics Institute (http://www.genenames.org).

b The gene trap vector in this clone inserted between the genes encoding SMN and DUSP5.

### Identification of a human cell line sensitive to ε-toxin

One difficulty in using MDCK cells to study the effects of ε-toxin on mammalian cells is the scarcity of reagents (*e.g.*, antibodies and shRNA) to canine proteins and genes. Such studies would be aided by the identification of a human cell line that is sensitive to the toxin. However, most cell lines tested appear resistant to ε-toxin, despite many being derived from organs and tissues that are susceptible to and damaged by ε-toxin *in vivo*. Other than MDCK cells, published reports identify only two alternative cell lines that are sensitive to ε-toxin [25], [39]. Like MDCK cells, both of these cell lines are derived from kidney: one is derived from mouse kidney, and the other is the human renal leiomyoblastoma cell line, G-402. In contrast to MDCK cells, G-402 cells are reported to require 160-times more ε-toxin to kill than MDCK cells [39]. We therefore sought to identify an alternative human cell line that might be more sensitive to ε-toxin in which to study the genes identified in our gene-trap selection.

**Figure 2:**
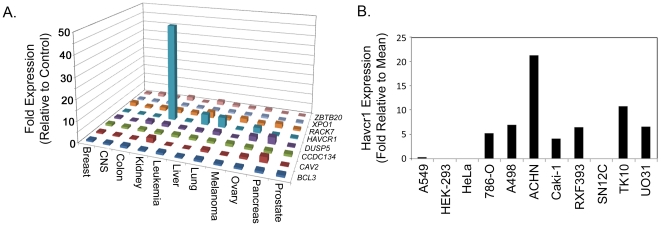
*HAVCR1* is more abundantly expressed in human kidney cells than in human cell lines resistant to ε-toxin. **A**. Gene expression data from a publicly-available microarray experiment were reported as fold increase over mean expression levels and included data for 8 genes listed in Table 1 [40], [41]. Data was sorted based on tissue of origin and the mean expression level of each gene was determined for each tissue. Results are expressed as the fold increase over the expression level observed in three control cell lines (A549, HEK-293, and HeLa) known to be resistant to ε-toxin. **B**. Gene expression data from a publicly-available microarray experiment reported as fold increase over mean expression levels among all 83 cell lines are displayed for three cell lines known to be resistant to ε-toxin (A549, HEK-293, and HeLa) and for 8 cell lines derived from human kidney with unknown sensitivity to ε-toxin [39].

We hypothesized that many human cell lines are resistant to ε-toxin-induced cytotoxicity because they fail to express one or more of the genes identified in the gene trap selection. To test this hypothesis, we used publicly-available microarray expression data from 83 human cell lines representing different tissues [40], [41]. Expression data was available for 8 of the genes listed in Table 1. Gene expression levels among cells with unknown sensitivity to ε-toxin were compared to the expression levels among cell lines known to be resistant to the toxin (HEK-293, HeLa, and A549) ([39] and data not shown). Results revealed one gene, *HAVCR1*, is expressed at higher levels in a number of human kidney cells than it is expressed in three human cell lines known to be resistant to ε-toxin (Figure 2).

**Figure 3:**
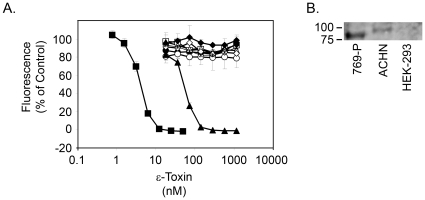
ACHN cells are sensitive to ε-toxin. **A**. Cell lines, including human kidney cell lines expressing HAVCR1 based on publicly-available microarray data or literature searches, were treated with serial dilutions of purified ε-toxin. The canine kidney cell line MDCK (▪) was included as a positive control; HeLa (◊) and HEK-293 (x) cells were included as negative controls. Other cell lines were 769-P (□), 786-O (•), A-498 (○), ACHN (▴), Caki-1 (▵), and G-402 (♦). Cell viability was assessed as described in the Materials and Methods. Results represent the mean and standard deviation of quadruplicate samples. **B**. Whole-cell lysates from ε-toxin-sensitive ACHN and ε-toxin-resistant 769-P and HEK-293 cells were prepared and equal volumes of samples were analyzed by immunoblot analysis using an anti-HAVCR1 antibody.

To identify one or more human kidney cell lines susceptible to ε-toxin, several kidney cell lines expected to express HAVCR1 were tested for toxin sensitivity. We found that one of the cell lines, ACHN, is sensitive to ε-toxin-induced cytotoxicity (Figure 3A), whereas G-402 and other cell lines examined were resistant to ε-toxin at the doses tested. Immunoblotting with an anti-HAVCR1 antibody was used to examine the expression of HAVCR1 among the cell lines tested. Results revealed that ACHN cells express a ∼100 kDa variant of HAVCR1 (Figure 3B). In contrast, cell lines resistant to ε-toxin express either no detectable HAVCR1 or a shorter, ∼90 kDa variant (Figure 3B and data not shown).

### Gene-specific knock-down with shRNAs reveals a role for HAVCR1 in ε-toxin-induced cytotoxicity

**Figure 4:**
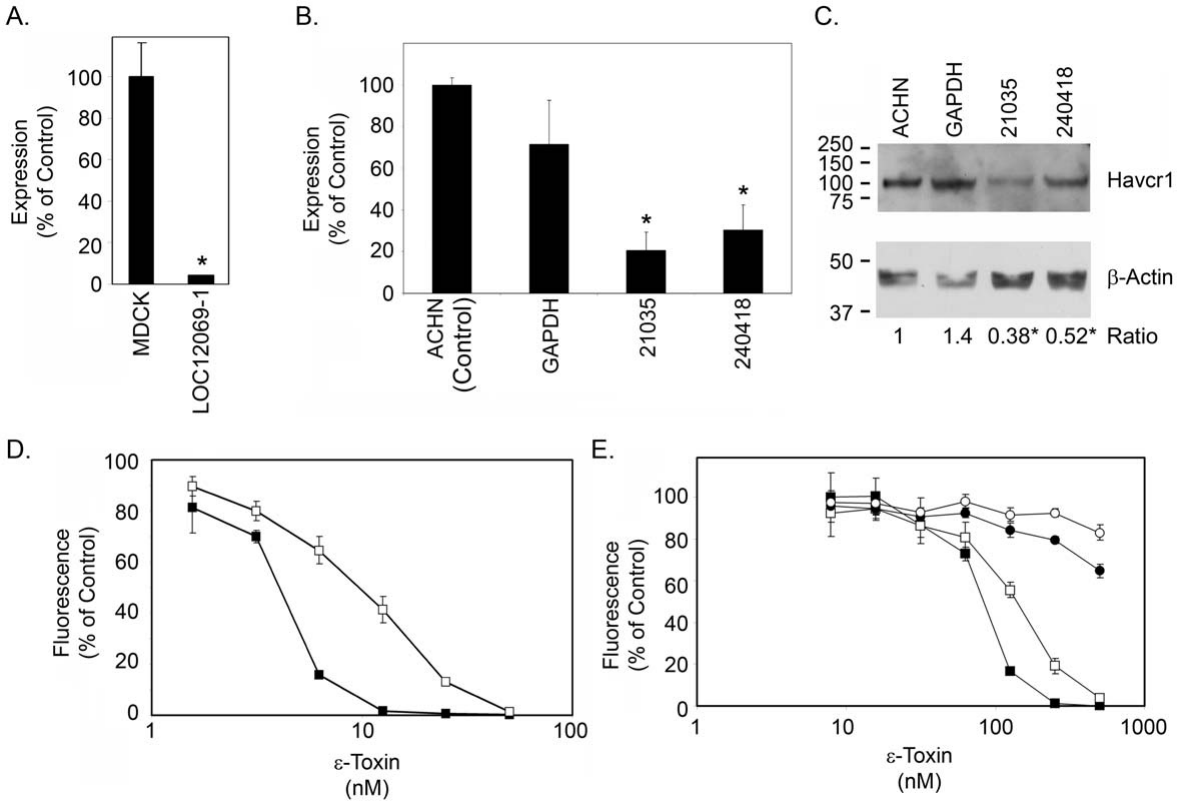
*HAVCR1* expression knockdown by shRNA. **A**. MDCK cells were stably transduced with a shRNA (LOC12069-1) targeting the *HAVCR1* transcript. Quantitative real-time PCR was used to determine the amount of *HAVCR1* transcript in the stably-transduced cell line compared to non-transduced MDCK cells. Results of quadruplicate samples are shown. The asterisk denotes results that are significantly different from non-transfected MDCK cells (p<0.05, ANOVA followed by Dunnett's post hoc test). **B**. ACHN cells were stably transfected with a shRNA to GAPDH or two shRNAs (21035 and 240418) targeting different regions of the *HAVCR1* transcript. Quantitative real-time PCR was used to determine the amount of *HAVCR1* transcript in the stably-transfected cell lines compared to non-transfected ACHN cells (control). Results summarize triplicate rt-PCR reactions from each of three independent RNA samples per cell line. Asterisks denote results that are significantly different from non-transfected ACHN cells (p<0.05, ANOVA followed by Dunnett's post hoc test). **C**. Whole cell lysates from non-transfected and stably-transfected ACHN cells were immunoblotted with an anti-HAVCR1 polyclonal antibody and with anti-β-actin as a control. The signal intensities from three different sets of lysates were quantified by densitometry and are expressed as the mean ratio of HAVCR1 to β-actin (normalized to non-transfected ACHN cells). Asterisks denote results that are significantly different from non-transfected ACHN cells (p<0.05, ANOVA followed by Dunnett's post hoc test). **D**. Non-transduced MDCK cells (▪) and MDCK cells stably-transduced with shRNA targeting *HAVCR1* (□, LOC12069-1) were treated with serial dilutions of purified ε-toxin. Cell viability was assessed as described in Materials and Methods. Results represent the mean and standard deviation of at least quadruplicate samples. The toxin dose needed to kill 50% of each cell line was calculated by non-linear regression analysis, and values were compared by Student's t-test. The MDCK cells transfected with *HAVCR1* shRNA required a greater amount of toxin to kill 50% of the cells than was required to kill the parental MDCK cells (P<0.0005). **E**. Cells (▪, ACHN; □, ACHN cells transfected with shRNA to GAPDH; •, ACHN cells transfected with *HAVCR1* shRNA 21035; ○, ACHN cells transfected with *HAVCR1* shRNA 240418) were treated with serial dilutions of purified ε-toxin. Cell viability was assessed as described in the Materials and Methods. Results represent the mean and standard deviation of quadruplicate samples. The toxin dose needed to kill 20% of cells was calculated by non-linear regression analysis and values were compared by ANOVA followed by Dunnett's *post hoc* test (the dose needed to kill 20% of the monolayer was determined rather than the dose needed to kill 50% as completing the dose response curve for cells transfected with *HAVCR1* shRNA would require considerable amounts of toxin). The ACHN cells transfected with *HAVCR1* shRNA required a greater amount of toxin to kill 20% of the cells than was required to kill 20% of the parental ACHN cells or cells transfected with *GAPDH* shRNA (P<0.05). The toxin dose required to kill 20% of cells transfected with *GAPDH* shRNA was not significantly different than the dose required to kill 20% of wild-type ACHN cells.

To provide further evidence that HAVCR1 contributes to ε-toxin-induced cytotoxicity, MDCK and ACHN cells were selected that stably express shRNA constructs directed against *HAVCR1* mRNA (Table 2). Despite repeated attempts, we were unable to obtain MDCK cells stably transfected with the LOC12069-2 shRNA. Analysis of *HAVCR1* mRNA by quantitative real-time PCR confirmed that cells expressing *HAVCR1*-specific shRNA exhibited reduced levels of *HAVCR1* mRNA (Fig. 4A and B), and immunoblotting confirmed that the cloned ACHN cells contained reduced levels of HAVCR1 protein (Figure 4C). Furthermore, MDCK and ACHN cells expressing reduced levels of *HAVCR1* mRNA were more resistant to ε-toxin than control cells (Fig. 4D and E). ACHN cells transfected with shRNA to GAPDH also exhibited a modest increase in resistance to the toxin compared to the non-transfected control (Fig. 4E), perhaps due to an off-target effect of the GAPDH shRNA or perhaps knocking down GAPDH expression has a modest effect on sensitivity to ε-toxin. The reduced toxin sensitivity observed in cells exhibiting reduced *HAVCR1* mRNA or HAVCR1 protein provides further evidence that HAVCR1 contributes to ε-toxin-induced cytotoxicity.

**Table 2:** HAVCR1 shRNAs.

Name	Sequence^a^	Location
*Canis familiaris*		
LOC12069-1	TCCCATTGCTTAGAAGAAATA	Exon 2
LOC12069-2	GGTTCAATGACATGAAACTCA	Exon 2
*Homo sapiens*		
V2LHS_21035	CTGTGTATAGTCAACCTCA	3′ UTR
V2LHS_240418	GCCAATACCACTAAAGGAA	Exon 6

a The sequence of the sense strand of the shRNA hairpin is provided.

### *HAVCR1* splice variants

**Figure 5:**
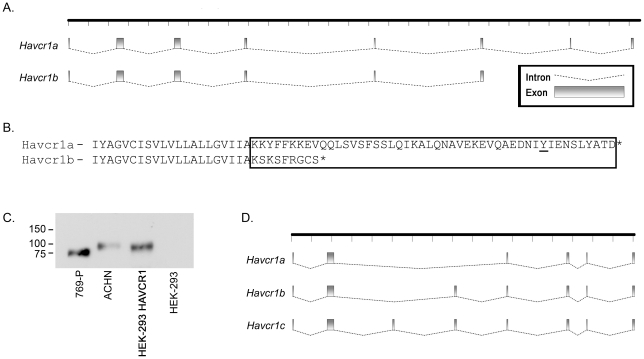
*HAVCR1* splice variants. **A**. Full-length cDNAs were cloned and sequenced from ε-toxin-sensitive ACHN cells (*HAVCR1a*) and from ε-toxin-resistant 769-P cells (*HAVCR1a* and *HAVCR1b*). The coding-exon structure for each transcript is illustrated. **B**. Partial amino acid sequences of the HAVCR1a and HAVCR1b proteins are shown; the putative cytoplasmic domains are boxed. A conserved tyrosine present in HAVCR1a, shown previously to become phosphorylated, is underlined [44], [45]. **C**. Whole-cell lysates were prepared from ε-toxin-sensitive ACHN, from ε-toxin-resistant 769-P and HEK-293 cells, and from HEK-293 cells transfected with a vector expressing HAVCR1a from ACHN cells and analyzed by immunoblot analysis using an anti-HAVCR1 antibody. **D**. Full-length cDNAs were cloned and sequenced from ε-toxin-sensitive MDCK cells. The coding-exon structures for the three transcripts are illustrated as in “A”.

We next examined the basis for the difference in the size of the HAVCR1 protein expressed by toxin-sensitive ACHN and toxin-resistant 769-P cells (Figure 3B). RNA was isolated from both cell lines, transcript ends were mapped by 5′- and 3′-RACE, and full-length cDNAs were prepared and sequenced. A single 3′ end to the open reading frame was identified in ε-toxin-sensitive ACHN cells, corresponding to the canonical HAVCR1 protein (*HAVCR1a*, Figure 5A). Two distinct 3′ ends to the open reading frame were identified in ε-toxin-resistant 769-P cells; one transcript (*HAVCR1a*) corresponded to the canonical sequence whereas the second, shorter transcript (*HAVCR1b*) expresses a previously un-described, truncated protein [42], [43]. Although the proteins encoded by both transcripts, HAVCR1a and HAVCR1b, are expected to have similar extracellular domains, the cytoplasmic domain of the HAVCR1a protein includes a tyrosine previously shown to undergo phosphorylation in response to ligand stimulation [44], [45]; HAVCR1b (the result of alternative splicing) includes a truncated cytoplasmic domain lacking the phosphorylation motif (Figure 5B).

To confirm that the *HAVCR1a* transcript cloned from ACHN cells expresses a protein similar in size to that identified by immunoblotting ACHN cells, the cDNA encoding *HAVCR1a* from ACHN cells was cloned into a mammalian expression vector and transfected into HEK-293 cells. Non-transfected HEK-293 cells express little, if any, HAVCR1 protein (Figures 3B and 5C), whereas HEK-293 cells transfected with *HAVCR1a* cDNA express a HAVCR1 protein similar in size to that expressed by toxin-sensitive ACHN cells (Figure 5C).

**Figure 6:**
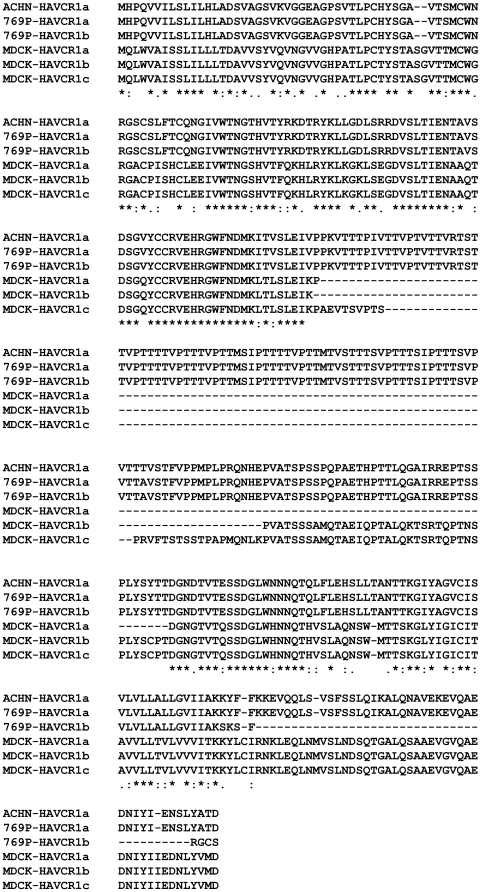
Amino acid sequence alignment of human and canine HAVCR1 splice variants. The predicted amino acid sequences of HAVCR1 proteins from human ACHN and 769-P cells and from canine MDCK cells were aligned using ClustalW. These sequences have been deposited in GenBank as accession numbers HQ412639 (HAVCR1a, ACHN cells), HQ412640 (Havcr1a, 769-P cells), HQ412641 (HAVCR1b, 769-P cells), HQ412642 (HAVCR1a, MDCK cells), HQ412643 (HAVCR1b, MDCK cells), and HQ412644 (HAVCR1c, MDCK cells).

Cloning and sequencing *HAVCR1* cDNA from MDCK cells led to the identification of 3 splice variants. The corresponding proteins vary in the length of an internal mucin-like domain (Figure 5C). Analysis of the amino acid sequences of *HAVCR1a* from ACHN and MDCK cells revealed that the two species of protein exhibit 50 to 60% amino acid identity within an amino-terminal IgV-like domain and the carboxy-terminal transmembrane and cytoplasmic domains; the internal mucin-like domain, though similar between the two species, varies considerably in length between the two species (Figure 6).

### ε-toxin binds to HAVCR1

**Figure 7:**
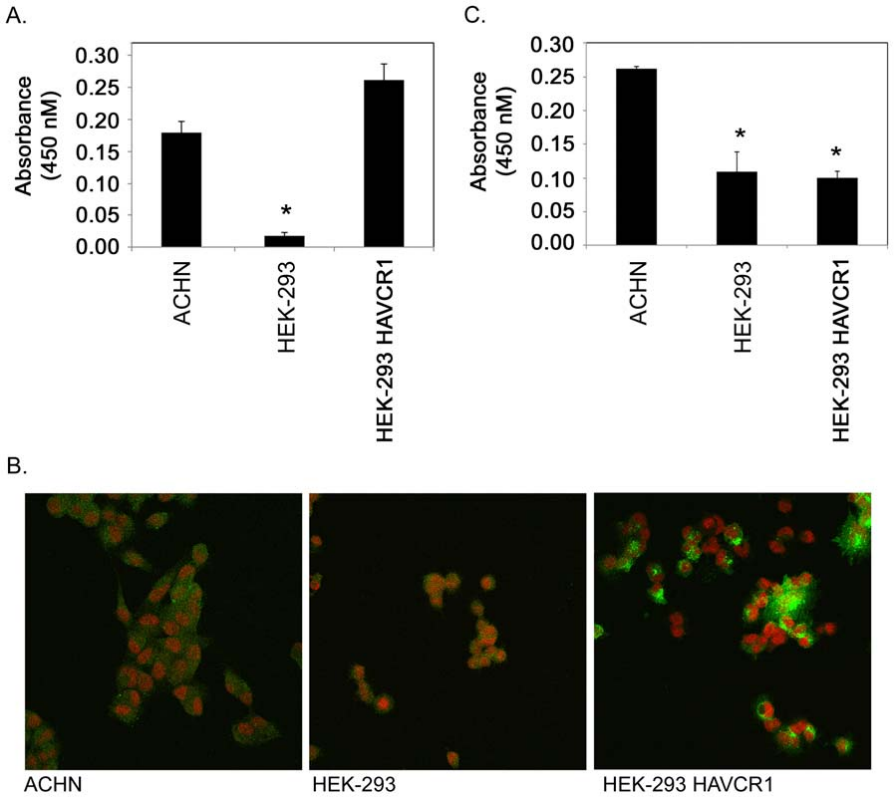
HAVCR1 expression in transfected HEK-293 cells. **A**. Expression of HAVCR1 in ACHN, HEK-293, and HEK-293 cells transfected with plasmid DNA expressing *HAVCR1a* from ACHN cells was detected by a cell-surface ELISA [43]. Representative results are presented as the mean and standard deviation of triplicate samples. The asterisk denotes results that are significantly less than the signal from toxin-sensitive ACHN cells (p<0.05, ANOVA followed by Dunnett's post hoc test). **B**. Expression of HAVCR1 on ACHN, HEK-293, and HEK-293 cells transfected with plasmid DNA expressing *HAVCR1a* from ACHN cells was detected by fluorescence microscopy as described in Materials and Methods. Green: HAVCR1; red: 7-AAD. **C**. Binding of ε-toxin to ACHN, HEK-293, and HEK-293 cells transfected with plasmid DNA expressing *HAVCR1a* from ACHN cells was detected by a cell-surface ELISA. Representative results are presented as the mean and standard deviation of triplicate samples. The asterisks denote results that are significantly less than the signal from toxin-sensitive ACHN cells (p<0.05, ANOVA followed by Dunnett's post hoc test).

HEK-293 cells are resistant to ε-toxin (Figure 3) and do not express detectable amounts of HAVCR1 protein (Figures 2 and 5C). To begin to examine the contribution of HAVCR1 to ε-toxin-induced-cytotoxicity, we sought to determine whether expression of HAVCR1 in HEK-293 cells would confer toxin sensitivity. HEK-293 cells were transfected with plasmid DNA expressing *HAVCR1a* from ε-toxin-sensitive ACHN cells. A cell-surface ELISA demonstrated that the HAVCR1 protein was present on the cell surface of the transfected cells at levels similar to that observed in toxin-sensitive ACHN cells (Figure 7A). Surface expression of HAVCR1 was also demonstrated by immunofluorescence microscopy (Figure 7B). However, the transfected HEK-293 cells remained resistant to the cytotoxic effects of ε-toxin (data not shown). These results indicate that expression of HAVCR1 in toxin-resistant HEK-293 cells is not sufficient to confer toxin sensitivity.

One possible role of HAVCR1 in ε-toxin-induced cytotoxicity is in promoting binding of the toxin to the cell surface. To examine the potential interaction between ε-toxin and HAVCR1, we measured toxin binding to cells. Using a cell-surface ELISA, ε-toxin binding was detected on toxin-sensitive ACHN cells; significantly less toxin bound to toxin-resistant HEK-293 cells (Figure 7C). However, expression of HAVCR1a in HEK-293 cells did not lead to a significant increase in the amount of toxin bound to the cells (Figure 7C). Thus, transfection of HEK-293 cells with *HAVCR1a* yielded a protein that bound specific antibodies to the receptor, but not to the toxin.

**Figure 8:**
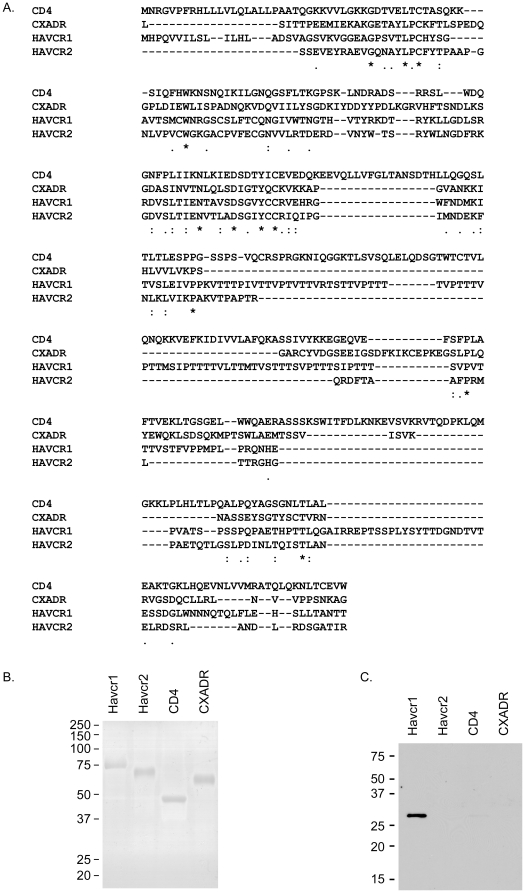
HAVCR1 binds to ε-toxin. **A**. The amino acid sequences of the recombinant extracellular domains of HAVCR1, HAVCR2 (TIM-3), CD4, CXADR were aligned using ClustalW. Despite similar tertiary structures, the proteins exhibit little primary amino acid sequence similarity. **B**. Equal amounts (0.5 µg) of the recombinant extracellular domains of HAVCR1, HAVCR2 (TIM-3), CD4, CXADR were separated by SDS-PAGE and the gel was stained with Simply Blue SafeStain (Invitrogen) demonstrating the purity of each protein preparation. **C**. Recombinant human extracellular domains of HAVCR1, CD4, CXADR, or HAVCR2 (TIM-3) were conjugated to paramagnetic beads as described in Materials and Methods. Purified ε-toxin was incubated with each conjugated bead preparation. The bound proteins were eluted by boiling in SDS, and detected by immunoblotting with anti- ε-toxin antiserum.

As a complementary approach to examine the potential interaction between ε-toxin and HAVCR1, we assessed the ability of the toxin to bind to the purified HAVCR1 extracellular domain. To identify suitable control proteins, we used the DALI server to compare the three-dimensional structure of the human HAVCR1 IgV-like domain to other human proteins [46], [47]. Three proteins (CD4, CXADR, and HAVCR2) were selected as the most similar structures to the HAVCR1 IgV-like domain. The calculated root mean square distances (RMSD) between HAVCR1 and CD4, CXADR, and HAVCR2 were 2.3, 1.9, and 1.3, respectively [46], [47], [48], [49], [50]. Although the tertiary structures of these proteins are similar, the primary amino acid sequences exhibit little similarity to the sequence of HAVCR1 (Figure 8A). Highly purified recombinant, human HAVCR1 extracellular domain or recombinant extracellular domains of CD4, CXADR, or HAVCR2 were conjugated to Dynabeads (Figure 8B). The protein conjugated beads then were mixed with purified ε-toxin Immunoblotting of eluted proteins indicated that ε-toxin bound to HAVCR1 coated beads, bound to a much lesser extent to CD4, but failed to bind to beads coated with CXADR or HAVCR2 (Figure 8C). These results indicate that ε-toxin can bind to the extracellular domain of HAVCR1.

## Discussion

The interactions between ε-toxin and mammalian cells leading to cell death are incompletely understood. Although evidence suggests that ε-toxin binds to a specific receptor on the surface of sensitive cells, the toxin also has been shown to form pores in protein-free liposomes [51]. In addition, the mechanism by which ε-toxin induces cell death is unclear. A previous study reported the isolation of spontaneous MDCK and G-402 cell variants with increased resistance to the ε-toxin, and two-dimensional PAGE revealed differences in the proteins expressed by sensitive and resistant cells [52]. However, the identities of these proteins and experiments confirming that these differences contributed to ε-toxin sensitivity were not reported. The present study confirms that host factors contribute to ε-toxin-induced cytotoxicity and identifies genes encoding several of these host factors. By identifying such host factors, this study illustrates that the lethal activity of the toxin is not simply a direct result of the pore-forming activity of the toxin.

Analysis of gene-expression microarray data led us to focus on one particular gene, *HAVCR1*, to use as an example to further validate the experimental approach. Cytotoxicity testing on cells expressing HAVCR1 led to the identification of a human cell line, ACHN, which is sensitive to ε-toxin. The identification of ε-toxin-sensitive ACHN cells provides an important experimental system with which to study the interaction between ε-toxin and human cells.

Results of the present study establish that HAVCR1 contributes to ε-toxin-induced cytotoxicity. Disruption of HAVCR1 expression, whether by gene-trap insertion or by RNA interference strategies in toxin-sensitive human and canine cell lines, led to increased resistance to ε-toxin-induced cytotoxicity. In addition, the toxin was found to bind with greater avidity to the extracellular domain of HAVCR1 than to structurally similar proteins, suggesting that HAVCR1 may serve as a receptor or co-receptor for the toxin. Whereas HAVCR1 also has been identified as a receptor for the hepatitis A virus [42], [43], it is possible that HAVCR1 participates in an alternate role. For example, HAVCR1 might participate in intracellular signal transduction in response to ε-toxin binding or to dysregulated ion flux through toxin pores [44], [45], or might participate in (or stabilize) protein-protein interactions that contribute to ε-toxin-induced cytotoxicity [53], [54].

Though we have demonstrated that HAVCR1 contributes to ε-toxin-induced cytotoxicity, expression of HAVCR1 in HEK-293 cells (a cell line that is naturally resistant to the toxin and which expresses undetected levels of HAVCR1) did not confer increased toxin binding or toxin sensitivity. Multiple explanations may account for this. For example, glycosylation is important for both ε-toxin binding to cells and for binding of hepatitis A virus to HAVCR1 [19], [55]. Thus, differences in the post-translational modification of HAVCR1 in ACHN and HEK-293 cells could alter toxin binding. Alternatively, HEK-293 cells may express one or more molecules that inhibit the binding or subsequent activity of the toxin. Such a cell-specific dominant phenotype has been observed previously in response to the cytocidal effect of tumor necrosis factor and in HIV virus replication [56], [57], [58]. Finally, HEK-293 cells may be deficient in one or more unidentified host factors (in addition to HAVCR1) contributing to ε-toxin-induced cytotoxicity. A similar complexity of interactions between a toxin and a host cell is illustrated by the identification of multiple factors contributing to the activity of the anthrax toxins [59], [60], [61], [62]. In support of these alternatives, analysis of publicly-available gene-expression microarray data reveals numerous genes expressed at higher levels in toxin-resistant HEK-293 cells than in toxin-sensitive ACHN cells ([40], [41]and data not shown). The same data also reveals genes expressed at higher levels in ACHN cells than in HEK-293 cells, including genes encoding proteins known to interact with HAVCR1 ([40], [41], [53], [54] and data not shown). We currently are determining whether these proteins, like HAVCR1, also contribute to ε-toxin-induced cytotoxicity.

Variability in HAVCR1 previously has been associated with susceptibility to allergic diseases [63], and also may be associated with susceptibility to infectious diseases [64], [65], [66], [67]. Whether ε-toxin has provided a selective pressure contributing to such variability in HAVCR1 is unclear. However, results of the present study suggest a correlation between the expression of a particular HAVCR1 splice variant and sensitivity to the ε-toxin. The recombinant HAVCR1 shown to bind ε-toxin coincides with the extracellular domain of the protein. The extracellular domains of the HAVCR1a and HAVCR1b variants we identified differ by a single amino acid (Figure 6), suggesting that the toxin either binds to both human isoforms of HAVCR1 characterized in this study, or that this single amino acid is critical for the toxin interaction. The region of least similarity between the HAVCR1a protein expressed by ε-toxin-sensitive ACHN cells and the shorter HAVCR1b protein identified in ε-toxin-resistant 769-P cells resides within the protein's cytoplasmic domain. The cytoplasmic domain in HAVCR1a includes a tyrosine previously shown to be subject to phosphorylation in response to relevant stimulation [44] and required for heterotypic protein-protein interactions [53]. A similar tyrosine is found in the cytoplasmic domain of the HAVCR1 protein variants identified in ε-toxin-sensitive MDCK cells. Further experiments are needed to identify additional cell lines, beyond ACHN, that express the HAVCR1a variant and to assess the sensitivities of such cell lines to ε-toxin.

This study presents the combined use of gene trap insertional mutagenesis and RNA interference to identify mammalian genes that contribute to the cytotoxic activity of a bacterial pore-forming toxin. Whereas previous studies using a similar approach have emphasized use of hypodiploid cell lines [34], [35], the present study demonstrates that a forward genetic approach may be employed using a common diploid cell line to perform a genome-wide survey identifying mammalian genes contributing to the activity of a bacterial protein toxin. Additional characterization of HAVCR1 and the other host factors identified in the present study is expected to provide insight into the mechanism of ε-toxin-induced cell death. For example, one of the genes identified and reported in this study was the gene encoding sphingomyelin synthase 2, whose product catalyzes the last step in the synthesis of sphingomyelin. A previous study has demonstrated that D609, an inhibitor of sphingomyelin synthase, inhibits ε-toxin-induced cytotoxicity [38]. Thus, the identification of this gene among our set of cells with increased resistance to the ε-toxin provides additional validity to the method. In addition, we also identified the gene encoding caveolin 2, in which shRNA knock down experiments validate a role for this protein in toxin activity (manuscript in preparation). Although caveolin 2, along with caveolin 1, is most frequently considered in its role in endocytosis of caveolae, multiple studies have reported being unable to detect internalization of ε-toxin into sensitive cells [21], [22], [24]. Insight into the role caveolin 2 plays might come from studies of another pore-forming toxin. For example, the *Staphylococcus aureus* α-toxin has been demonstrated to bind to caveolin 1, and mutant α-toxins that are disrupted in the ability to bind caveolin 1 are non-hemolytic [68], [69], [70]. Further studies are needed to determine the precise role caveolin 2 plays in ε-toxin-induced cytotoxicity. Many of the other genes identified in the present study appear to be involved in intracellular signal transduction and gene transcription. Additional studies are in progress to confirm a role for these genes in ε-toxin-induced cytotoxicity and to further explore the signal-transduction pathways and transcriptional response of intoxicated cells. The data presented in this paper indicate that mammalian genes may be important participants to the activity of bacterial toxins, and may serve as targets for therapeutics.

## Materials and Methods

### Cell culture

MDCK, HeLa, ACHN, HEK-293, and A-498 cells were cultured in MEM. 769-P and 786-O cells were cultured in RPMI. Caki-1 and G-402 cells were cultured in McCoy's 5A. All cell lines were obtained from ATCC, and all culture media were supplemented with 10% serum (Fetal Clone III, HyClone).

### Gene trap library construction

The U3neoSV1 retrovirus vector [36] was obtained from Zirus, Inc. (Buford, GA). Toxin-sensitive MDCK cells were infected in T75 flasks at 37°C for 1 hour with U3neoSV1 (MOI = 0.1) in the presence of 4 µg/ml Polybrene (Sigma, St. Louis, MO). Subsequently, the medium was changed and the cells were grown overnight at 37°C. On the following day, G418 selection was initiated and cells were grown to confluence.

### Generation of ε-toxin-resistant MDCK cells from the gene trap library

MDCK cells transfected with the gene trap vector were plated in nine 100 mm dishes (approximately 3.3×10^6^ cells per dish) in Leibovitz's L-15 medium. Toxin was added to a final concentration of 20 nM, and the treated cells were incubated at 37°C for 16 hours. The toxin-containing medium was replaced with fresh medium and surviving cells were cultured for 2 days. Medium then was replaced with fresh Leibovitz's L-15 containing 35 nM ε-toxin, and the cells were incubated for 16 hours. The toxin-containing medium was replaced with fresh medium and surviving cells were cultured for 3 days. Medium then was replaced with fresh Leibovitz's L-15 containing 50 nM ε-toxin, and the cells were incubated for 16 hours. Medium then was replaced with fresh Leibovitz's L-15 (with no added toxin) and cells that survived exposure to ε-toxin were allowed to grow 7 days before clonal populations were isolated. Clonal populations were isolated by limiting dilution into multiwell plates, followed by microscopic examination to identify wells receiving a single cell. These single cells then were propagated to provide sufficient cells for analysis. The cloning of a single integrated gene-trap vector from each of the toxin-resistant cell lines confirmed the clonal nature of the cell lines.

### U3neoSV1 shuttle vector rescue, sequencing, and analysis

Cellular DNAs from clonal toxin-resistant cell lines were extracted using a QIAamp DNA Blood Maxi kit (QIAGEN, Inc., Valencia, CA). Shuttle vectors and genomic DNA flanking the integration site were recovered by digestion of the genomic DNA (150 mg/ml) with EcoRI or BamHI, self-ligation, transformation into *Escherichia coli*, and selection on Luria broth agar containing 100 µg per ml carbenicillin (Sigma). Individual colonies were amplified, and recovered genomic DNAs were sequenced using primers annealing to the gene-trap shuttle vector. Sequencing reactions were performed using an ABI BigDye Terminator cycle sequencing kit, and sequences were analyzed using an ABI 3100 genetic analyzer (Applied Biosystems, Foster City, CA). The sequences of the sequencing primers are available upon request. Genomic sequences obtained from shuttle clones were analyzed by the RepeatMasker web server, followed by nucleotide-nucleotide BLAST searches against the National Center for Biotechnology Information databases.

### Transfections and transductions

Plasmid DNAs encoding shRNAs were transfected into ACHN cells using Lipofectamine 2000 (Invitrogen). Pseudoviral particles encoding shRNAs were purchased from Sigma-Aldrich and transduced into MDCK cells. The sequences and locations of shRNAs are listed in Table 2. Plasmid DNA (pCMV-Script, Stratagene) encoding the HAVCR1 protein from ACHN cells was transfected into HEK-293 cells using Lipofectamine LTX (Invitrogen).

### Real-time quantitative PCR

Cells were treated with RNALater (Ambion) according to the manufacturer's instructions. Total cellular RNA was isolated from the treated cells with TriZol reagent (Invitrogen). Complementary DNA (cDNA) was prepared from 2 µg total RNA using Omniscript RT (Qiagen). Quantitative real-time PCR was performed using gene-specific primer sets from Applied Biosystems and a StepOne Plus instrument (Applied Biosystems). A primer set specific to 18S rRNA was used as control. Results were analyzed by comparing *C*_T_ values [71].

### ε-toxin purification and cytotoxicity assays

Previous studies have demonstrated that native and recombinant ε-toxin produced in *E. coli* exhibit similar specific activities in standard tissue-culture based assays of toxin activity [20], [37], [72], [73], [74], [75]. Recombinant ε-prototoxin was expressed in *E. coli* K12 strain NovaBlue (DE3) (Novagen), and purified essentially as described [74], [75]. The cells were collected by centrifugation, resuspended in 5% culture volume of B-PER Bacterial Protein Extraction Reagent (Pierce) supplemented with Complete Mini protease inhibitor cocktail (EDTA-free, Roche), and mixed for 10 minutes at room temperature. Omnicleave nuclease (Epicentre) was added to reduce the viscosity of the samples. The cell debris was pelleted, and the supernatant was recovered. The B-PER extracted material was diluted four-fold with water, and applied to a Q-Sepharose column. The ε-prototoxin-containing flow-through material was collected and applied to a Ni-NTA affinity column (Qiagen). The Ni-NTA column was washed with a buffer comprised of 50 mM sodium phosphate, 300 mM sodium chloride, and 20 mM imidazole (pH 8.0), and the ε-prototoxin was eluted in a buffer comprised of 50 mM sodium phosphate, 300 mM sodium chloride, and 250 mM imidazole (pH 8.0). The identification of the ε-prototoxin in the purified sample was confirmed by immunoblotting with ε-toxin-specific monoclonal antibody. Protein concentrations were determined using micro-BCA (Pierce).

The ε-prototoxin can be cleaved with trypsin to remove short peptides from both the amino- and carboxy-terminal ends of the protein to yield the active ε-toxin [20], [74], [75], [76], [77]. Trypsin-coated agarose beads (Pierce) were washed and resuspended in 5 mM Tris, pH 7.5. Preparations containing the ε-prototoxin were incubated with trypsin-agarose at 37°C for 60 minutes, the trypsin-coated beads were removed by centrifugation, and residual trypsin was inhibited by Complete Mini protease inhibitor cocktail (Roche). Conversion of the ε-prototoxin to ε-toxin was assessed based on SDS-PAGE and immunoblotting with anti- ε-toxin antibodies.

Cytotoxicity was determined based on assessing cellular metabolic activity as described [75]. Cells in Leibovitz's L-15 medium supplemented with 10% fetal bovine serum were added to multiwell plates (5×10^3^ cells per well in 384-well dishes) and incubated at 37°C. Following cell attachment, ε-toxin was added, and the cells then were incubated at 37°C for 16 hours. Cytotoxicity was determined by treating cells with resazurin (CellTiter Blue, Promega) at 37°C for 4 hours. Fluorescence at 590 nm was measured following excitation at 560 nm using a BioTek FLx800 plate reader. Results were normalized to the fluorescent signal from cells incubated in the absence of toxin (100%) and in 0.1% Triton X-100 (0%).

### RACE analysis

The 5′- and 3′-ends of HAVCR1 transcripts in ACHN and 769-P cells were determined by RACE analysis using First Choice RLM RACE (Ambion) and DNA sequences were determined (GenHunter).

### Bead binding assay

Recombinant proteins (R & D Systems) were conjugated to tosyl-activated Dynabeads according to the manufacturer's instructions (Invitrogen). To compare the efficiency of protein conjugation to the beads, the protein concentration of an aliquot of each bead preparation was determined using the micro BCA assay (Pierce); the amount of bound protein varied by less than 5%. For capturing ε-toxin from solution, coated beads in Tris-buffered saline supplemented with 0.1% tween-20 and 0.1% BSA were mixed with purified ε-toxin. Beads were incubated with the toxin at room temperature for 1 hour, with agitation. The beads then were captured, washed 3 times in Tris-buffered saline containing 0.1% tween-20. The washed beads then were heated in SDS-sample buffer to elute bound toxin, and samples were analyzed by SDS-PAGE followed by immunoblotting with anti- ε-toxin antiserum.

### Immunological reagents

Anti-*Clostridium perfringens* C/D (Boerhinger-Ingelheim) equine antiserum was used to detect purified ε-toxin in immunoblots; mouse monoclonal antibody 4D7 was used to detect ε-toxin bound to cells [78]. Mouse monoclonal antibody to human HAVCR1 (Tim-1, R & D Systems), and rabbit polyclonal antibody to β-actin (Abcam) were used as primary antibodies for the detection of HAVCR1, and β-actin, respectively. HRP-conjugated donkey anti-rabbit (GE Healthcare), donkey anti-goat (Abcam), and rabbit anti-horse (Sigma-Aldrich) were used as secondary antibodies. SuperSignal West Pico and SuperSignal West Femto (Thermo Scientific) were used for enhanced chemiluminescent detection.

### Cell surface ELISA

A cell-surface ELISA was used to detect surface expression of HAVCR1 [43]. Cells were detached from tissue culture flasks using 0.2% EDTA in 0.9% NaCl. Cells were transferred to 96 well V-bottom plates at 3×10^5^ cells per well. Cells then were fixed with 0.08% glutaraldehyde for 15 minutes at room temperature. Cells were washed with PBS, and incubated with PBS containing 5% BSA for 30 minutes at room temperature. HAVCR1 was detected with mouse anti-HAVCR1 monoclonal antibody (R & D Systems) for one hour at room temperature and horseradish peroxidase-labeled anti-mouse IgG (45 minutes at room temperature) followed by TMB substrate (Pierce). The signal from control cells treated with the secondary antibody only was subtracted.

A similar approach was used to detect ε-toxin binding to cells. Fixed cells were washed with PBS, and toxin was added to 625 nM for 15 minutes at 37°C. After removal of unbound toxin, cells were incubated with PBS containing 5% BSA for 30 minutes at room temperature. Bound ε-toxin was detected with mouse anti- ε-toxin monoclonal antibody 4D7 [78]. Cells were incubated with anti- ε-toxin antibody for one hour at room temperature and horseradish peroxidase-labeled anti-mouse IgG (45 minutes at room temperature) followed by TMB substrate (Pierce). The signal from control cells, in the absence of added toxin was subtracted.

### Fluorescence Microscopy

Cells were grown on an 8-well chamber slide (Nunc) to 20% confluency. Cells were washed with warm HBSS (0.137 M NaCl, 5.4 mM KCl, 0.25 mM Na_2_HPO_4_, 0.44 mM KH_2_PO_4_, 1.3 mM CaCl_2_, 1.0 mM MgSO_4_, 4.2 mM NaHCO_3_) and incubated with anti-HAVCR1 mouse monoclonal antibody (R&D Systems) in MEM with 10% FBS for 1 hour at 37°C. The media was removed from each well and cells subsequently were fixed in 4% formaldehyde in HBSS for 10 minutes at 37°C. Cells were washed 3 times with warm HBSS and permeabilized by incubation with 0.25% Triton-X 100 (Sigma-Aldrich) in HBSS for 5 minutes at 37°C. Cells were washed with warm HBSS and subsequently blocked for 30 minutes at 37°C with 10% goat serum (Sigma-Aldrich) in HBSS. The cells were washed 3 times with HBSS, and incubated in the dark with Alexa Fluor 488-conjugated goat anti-murine IgG (Invitrogen) diluted in MEM with 10% FBS for 1 hour at 4°C. Cells were washed 3 times with HBSS then each well was incubated with 7-AAD nuclear stain (BD Pharmingen) for 15 minutes at 37°C in the dark. Cells were washed 3 times in HBSS, the chambers were removed, and a coverslip was mounted with Fluoro-Gel (Electron Microscopy Sciences). Imaging was performed on a Zeiss LSM 510 inverted confocal microscope.

### Select agent

Plasmid DNA capable of expressing the ε-prototoxin (or ε-toxin) is considered a select agent by the U.S. Department of Health and Human Services.

## References

[1] Smith LDS, Williams BL (1984). The Pathogenic Anaerobic Bacteria.

[2] Goldstein J, Morris WE, Loidl CF, Tironi-Farinatti C, McClane BA (2009). Clostridium perfringens epsilon toxin increases the small intestinal permeability in mice and rats.. PLoS One.

[3] Adamson RH, Ly JC, Fernandez-Miyakawa M, Ochi S, Sakurai J (2005). Clostridium perfringens epsilon-toxin increases permeability of single perfused microvessels of rat mesentery.. Infect Immun.

[4] Soler-Jover A, Dorca J, Popoff MR, Gibert M, Saura J (2007). Distribution of Clostridium perfringens epsilon toxin in the brains of acutely intoxicated mice and its effect upon glial cells.. Toxicon.

[5] Ghabriel MN, Zhu C, Reilly PL, Blumbergs PC, Manavis J (2000). Toxin-induced vasogenic cerebral oedema in a rat model.. Acta Neurochir.

[6] Worthington RW, Mulders MS (1975). Effect of Clostridium perfringens epsilon toxin on the blood brain barrier of mice.. Onderstepoort J Vet Res.

[7] Nagahama M, Sakurai J (1991). Distribution of labeled Clostridium perfringens epsilon toxin in mice.. Toxicon.

[8] Soler-Jover A, Blasi J, de Aranda IG, Navarro P, Gibert M (2004). Effect of Epsilon Toxin-GFP on MDCK Cells and Renal Tubules In Vivo.. J Histochem Cytochem.

[9] Fernandez-Miyakawa ME, Sayeed S, Fisher DJ, Poon R, Adams V (2007). Development and application of a mouse oral challenge model for studying Clostridium perfringens type D infection.. Infect Immun.

[10] Uzal FA, Kelly WR, Morris WE, Assis RA (2002). Effects of intravenous injection of Clostridium perfringens type D epsilon toxin in calves.. J Comp Pathol.

[11] Uzal FA, Kelly WR, Morris WE, Bermudez J, Baison M (2004). The pathology of peracute experimental Clostridium perfringens type D enterotoxemia in sheep.. J Vet Diagn Invest.

[12] Nagahama M, Kobayashi K, Ochi S, Sakurai J (1991). Enzyme-linked immunosorbent assay for rapid detection of toxins from Clostridium perfringens.. FEMS Microbiol Lett.

[13] Sidorenko GI (1967). Data on the distribution of Clostridium perfringens in the environment of man. Communication 1.. J Hyg Epidemiol Microbiol Immunol.

[14] Gleeson-White MH, Bullen JJ (1955). Clostridium welchii epsilon toxin in the intestinal contents of man.. Lancet.

[15] Kohn J, Warrack GH (1955). Recovery of Clostridium welchii type D from man.. Lancet.

[16] Miller C, Florman S, Kim-Schluger L, Lento P, De La Garza J (2004). Fulminant and fatal gas gangrene of the stomach in a healthy live liver donor.. Liver Transpl.

[17] Morinaga G, Nakamura T, Yoshizawa J, Nishida S (1965). Isolation of Clostridium perfringens Type D from a Case of Gas Gangrene.. J Bacteriol.

[18] Buxton D (1976). Use of horseradish peroxidase to study the antagonism of Clostridium welchii (Cl. perfringens) type D epsilon toxin in mice by the formalinized epsilon prototoxin.. J Comp Pathol.

[19] Nagahama M, Sakurai J (1992). High-affinity binding of Clostridium perfringens epsilon-toxin to rat brain.. Infect Immun.

[20] Miyata S, Matsushita O, Minami J, Katayama S, Shimamoto S (2001). Cleavage of a C-terminal peptide is essential for heptamerization of Clostridium perfringens epsilon-toxin in the synaptosomal membrane.. J Biol Chem.

[21] Nagahama M, Ochi S, Sakurai J (1998). Assembly of Clostridium perfringens epsilon-toxin on MDCK cell membrane.. J Nat Toxins.

[22] Petit L, Gibert M, Gillet D, Laurent-Winter C, Boquet P (1997). Clostridium perfringens epsilon-toxin acts on MDCK cells by forming a large membrane complex.. J Bacteriol.

[23] Petit L, Maier E, Gibert M, Popoff MR, Benz R (2001). Clostridium perfringens epsilon toxin induces a rapid change of cell membrane permeability to ions and forms channels in artificial lipid bilayers.. J Biol Chem.

[24] Lindsay CD (1996). Assessment of aspects of the toxicity of Clostridium perfringens epsilon-toxin using the MDCK cell line.. Hum Exp Toxicol.

[25] Chassin C, Bens M, de Barry J, Courjaret R, Bossu JL (2007). Pore-forming epsilon toxin causes membrane permeabilization and rapid ATP depletion-mediated cell death in renal collecting duct cells.. Am J Physiol Renal Physiol.

[26] Gonzalez MR, Bischofberger M, Pernot L, van der Goot FG, Freche B (2008). Bacterial pore-forming toxins: the (w)hole story?. Cell Mol Life Sci.

[27] Bischof LJ, Kao CY, Los FC, Gonzalez MR, Shen Z (2008). Activation of the unfolded protein response is required for defenses against bacterial pore-forming toxin in vivo.. PLoS Pathog.

[28] Gurcel L, Abrami L, Girardin S, Tschopp J, van der Goot FG (2006). Caspase-1 activation of lipid metabolic pathways in response to bacterial pore-forming toxins promotes cell survival.. Cell.

[29] Huffman DL, Abrami L, Sasik R, Corbeil J, van der Goot FG (2004). Mitogen-activated protein kinase pathways defend against bacterial pore-forming toxins.. Proc Natl Acad Sci U S A.

[30] Bellier A, Chen CS, Kao CY, Cinar HN, Aroian RV (2009). Hypoxia and the hypoxic response pathway protect against pore-forming toxins in C. elegans.. PLoS Pathog.

[31] Zhang X, Candas M, Griko NB, Taussig R, Bulla LA (2006). A mechanism of cell death involving an adenylyl cyclase/PKA signaling pathway is induced by the Cry1Ab toxin of Bacillus thuringiensis.. Proc Natl Acad Sci U S A.

[32] Skals M, Jorgensen NR, Leipziger J, Praetorius HA (2009). Alpha-hemolysin from Escherichia coli uses endogenous amplification through P2X receptor activation to induce hemolysis.. Proc Natl Acad Sci U S A.

[33] Soletti RC, Alves T, Vernal J, Terenzi H, Anderluh G Inhibition of MAPK/ERK, PKC and CaMKII signaling blocks cytolysin-induced human glioma cell death.. Anticancer Res.

[34] Banks DJ, Bradley KA (2007). SILENCE: a new forward genetic technology.. Nat Methods.

[35] Carette JE, Guimaraes CP, Varadarajan M, Park AS, Wuethrich I (2009). Haploid genetic screens in human cells identify host factors used by pathogens.. Science.

[36] Osipovich AB, White-Grindley EK, Hicks GG, Roshon MJ, Shaffer C (2004). Activation of cryptic 3′ splice sites within introns of cellular genes following gene entrapment.. Nucleic Acids Res.

[37] Payne DW, Williamson ED, Havard H, Modi N, Brown J (1994). Evaluation of a new cytotoxicity assay for Clostridium perfringens type D epsilon toxin.. FEMS Microbiol Lett.

[38] Shimamoto S, Tamai E, Matsushita O, Minami J, Okabe A (2005). Changes in Ganglioside Content Affect the Binding of Clostridium perfringens Epsilon-Toxin to Detergent-Resistant Membranes of Madin-Darby Canine Kidney Cells.. Microbiol Immunol.

[39] Shortt SJ, Titball RW, Lindsay CD (2000). An assessment of the in vitro toxicology of Clostridium perfringens type D epsilon-toxin in human and animal cells.. Hum Exp Toxicol.

[40] Su AI, Wiltshire T, Batalov S, Lapp H, Ching KA (2004). A gene atlas of the mouse and human protein-encoding transcriptomes.. Proc Natl Acad Sci U S A.

[41] Wu C, Orozco C, Boyer J, Leglise M, Goodale J (2009). BioGPS: an extensible and customizable portal for querying and organizing gene annotation resources.. Genome Biol.

[42] Kaplan G, Totsuka A, Thompson P, Akatsuka T, Moritsugu Y (1996). Identification of a surface glycoprotein on African green monkey kidney cells as a receptor for hepatitis A virus.. Embo J.

[43] Feigelstock D, Thompson P, Mattoo P, Zhang Y, Kaplan GG (1998). The human homolog of HAVcr-1 codes for a hepatitis A virus cellular receptor.. J Virol.

[44] de Souza AJ, Oak JS, Jordanhazy R, DeKruyff RH, Fruman DA (2008). T cell Ig and mucin domain-1-mediated T cell activation requires recruitment and activation of phosphoinositide 3-kinase.. J Immunol.

[45] de Souza AJ, Oriss TB, O'Malley KJ, Ray A, Kane LP (2005). T cell Ig and mucin 1 (TIM-1) is expressed on in vivo-activated T cells and provides a costimulatory signal for T cell activation.. Proc Natl Acad Sci U S A.

[46] Holm L, Sander C (1993). Protein structure comparison by alignment of distance matrices.. J Mol Biol.

[47] Santiago C, Ballesteros A, Tami C, Martinez-Munoz L, Kaplan GG (2007). Structures of T Cell immunoglobulin mucin receptors 1 and 2 reveal mechanisms for regulation of immune responses by the TIM receptor family.. Immunity.

[48] Cao E, Zang X, Ramagopal UA, Mukhopadhaya A, Fedorov A (2007). T cell immunoglobulin mucin-3 crystal structure reveals a galectin-9-independent ligand-binding surface.. Immunity.

[49] Ryu SE, Truneh A, Sweet RW, Hendrickson WA (1994). Structures of an HIV and MHC binding fragment from human CD4 as refined in two crystal lattices.. Structure.

[50] Seiradake E, Lortat-Jacob H, Billet O, Kremer EJ, Cusack S (2006). Structural and mutational analysis of human Ad37 and canine adenovirus 2 fiber heads in complex with the D1 domain of coxsackie and adenovirus receptor.. J Biol Chem.

[51] Nagahama M, Hara H, Fernandez-Miyakawa M, Itohayashi Y, Sakurai J (2006). Oligomerization of Clostridium perfringens epsilon-Toxin Is Dependent upon Membrane Fluidity in Liposomes.. Biochemistry.

[52] Beal DR, Titball RW, Lindsay CD (2003). The development of tolerance to Clostridium perfringens type D epsilon-toxin in MDCK and G-402 cells.. Hum Exp Toxicol.

[53] Kuehn EW, Hirt MN, John AK, Muehlenhardt P, Boehlke C (2007). Kidney injury molecule 1 (Kim1) is a novel ciliary molecule and interactor of polycystin 2.. Biochem Biophys Res Commun.

[54] Kotsis F, Nitschke R, Boehlke C, Bashkurov M, Walz G (2007). Ciliary calcium signaling is modulated by kidney injury molecule-1 (Kim1).. Pflugers Arch.

[55] Thompson P, Lu J, Kaplan GG (1998). The Cys-rich region of hepatitis A virus cellular receptor 1 is required for binding of hepatitis A virus and protective monoclonal antibody 190/4.. J Virol.

[56] Song C, Aiken C (2007). Analysis of human cell heterokaryons demonstrates that target cell restriction of cyclosporine-resistant human immunodeficiency virus type 1 mutants is genetically dominant.. J Virol.

[57] Nophar Y, Holtmann H, Ber R, Wallach D (1988). Dominance of resistance to the cytocidal effect of tumor necrosis factor in heterokaryons formed by fusion of resistant and sensitive cells.. J Immunol.

[58] Munk C, Brandt SM, Lucero G, Landau NR (2002). A dominant block to HIV-1 replication at reverse transcription in simian cells.. Proc Natl Acad Sci U S A.

[59] Scobie HM, Rainey GJ, Bradley KA, Young JA (2003). Human capillary morphogenesis protein 2 functions as an anthrax toxin receptor.. Proc Natl Acad Sci U S A.

[60] Bradley KA, Mogridge J, Mourez M, Collier RJ, Young JA (2001). Identification of the cellular receptor for anthrax toxin.. Nature.

[61] Abrami L, Kunz B, Deuquet J, Bafico A, Davidson G (2008). Functional interactions between anthrax toxin receptors and the WNT signalling protein LRP6.. Cell Microbiol.

[62] Wei W, Lu Q, Chaudry GJ, Leppla SH, Cohen SN (2006). The LDL receptor-related protein LRP6 mediates internalization and lethality of anthrax toxin.. Cell.

[63] Graves PE, Siroux V, Guerra S, Klimecki WT, Martinez FD (2005). Association of atopy and eczema with polymorphisms in T-cell immunoglobulin domain and mucin domain-IL-2-inducible T-cell kinase gene cluster in chromosome 5 q 33.. J Allergy Clin Immunol.

[64] Nakajima T, Wooding S, Satta Y, Jinnai N, Goto S (2005). Evidence for natural selection in the HAVCR1 gene: high degree of amino-acid variability in the mucin domain of human HAVCR1 protein.. Genes Immun.

[65] Genovese G, Friedman DJ, Ross MD, Lecordier L, Uzureau P Association of trypanolytic ApoL1 variants with kidney disease in African Americans.. Science.

[66] Pier GB, Grout M, Zaidi T, Meluleni G, Mueschenborn SS (1998). Salmonella typhi uses CFTR to enter intestinal epithelial cells.. Nature.

[67] Schroeder SA, Gaughan DM, Swift M (1995). Protection against bronchial asthma by CFTR delta F508 mutation: a heterozygote advantage in cystic fibrosis.. Nat Med.

[68] Pany S, Krishnasastry MV (2007). Aromatic residues of Caveolin-1 binding motif of alpha-hemolysin are essential for membrane penetration.. Biochem Biophys Res Commun.

[69] Vijayvargia R, Kaur S, Sangha N, Sahasrabuddhe AA, Surolia I (2004). Assembly of alpha-hemolysin on A431 cells leads to clustering of Caveolin-1.. Biochem Biophys Res Commun.

[70] Pany S, Vijayvargia R, Krishnasastry MV (2004). Caveolin-1 binding motif of alpha-hemolysin: its role in stability and pore formation.. Biochem Biophys Res Commun.

[71] Livak KJ, Schmittgen TD (2001). Analysis of relative gene expression data using real-time quantitative PCR and the 2(−Delta Delta C(T)) Method.. Methods.

[72] Miyata S, Minami J, Tamai E, Matsushita O, Shimamoto S (2002). Clostridium perfringens epsilon-toxin forms a heptameric pore within the detergent-insoluble microdomains of Madin-Darby canine kidney cells and rat synaptosomes.. J Biol Chem.

[73] Oyston PC, Payne DW, Havard HL, Williamson ED, Titball RW (1998). Production of a non-toxic site-directed mutant of Clostridium perfringens epsilon-toxin which induces protective immunity in mice.. Microbiology.

[74] Pelish TM, McClain MS (2009). Dominant-negative inhibitors of the clostridium perfringens {epsilon}-toxin.. J Biol Chem.

[75] Lewis M, Weaver CD, McClain MS (2010). Identification of Small Molecule Inhibitors of Clostridium perfringens epsilon-Toxin Cytotoxicity Using a Cell-Based High-Throughput Screen.. Toxins (Basel).

[76] McClain MS, Cover TL (2007). Functional Analysis of Neutralizing Antibodies against Clostridium perfringens Epsilon-Toxin.. Infect Immun.

[77] Minami J, Katayama S, Matsushita O, Matsushita C, Okabe A (1997). Lambda-toxin of Clostridium perfringens activates the precursor of epsilon-toxin by releasing its N- and C-terminal peptides.. Microbiol Immunol.

[78] Hauer PJ, Clough NE (1999). Development of monoclonal antibodies suitable for use in antigen quantification potency tests for clostridial veterinary vaccines.. Dev Biol Stand.

